# Six-week grit training course reduces school bullying: A quasi-experimental study

**DOI:** 10.3389/frcha.2022.1045808

**Published:** 2023-01-30

**Authors:** Lei Wang, Qianqian Chen, Zhenzhen Peng, Caixia Ye, Xinyi Zhou

**Affiliations:** ^1^ Department of Psychology, Wuhan Sport University, Wuhan, China; ^2^ Graduate School, Wuhan Sport University, Wuhan, China; ^3^ Department of Medicine, Wuhan City College, Wuhan, China; ^4^ Mental Health Education and Counseling Center, Wudang Middle School Affiliated to Central China Normal University, Shiyan, China

**Keywords:** cyberbullying, grit training courses, intervention, middle school students, school bullying

## Abstract

**Objective:**

To investigate the effectiveness of intervention programs in reducing school bullying, a 6-week series of grit training courses was designed and developed.

**Methods:**

Using a quasi-experimental design, 163 middle school students were selected as the experimental group and 201 middle school students as the control group to test the implementation effect of the grit training course.

**Results:**

The intervention program significantly reduced the traditional bullying behavior of the bullies and the traditional victimization of victims.

**Conclusion:**

The 6-week grit course has produced positive results in reducing school bullying. In the future, school-based courses with the theme of grit can be designed to achieve the purpose of preventing school bullying by improving the positive qualities of individuals.

## Introduction

School bullying is a growing concern in society. In bullying, regardless of the role of the participant, the occurrence of bullying can cause severe trauma to the individual. Individuals who play the role of the bully for a long time have a greatly increased probability of committing violent criminal acts in the future (1), threatening social stability. Individuals who have been bullied will experience obvious negative effects, such as psychological pressure, anxiety and depression due to fear, even personality disorders (2) and suicidal behaviors (3). Therefore, it is important to pay attention to school bullying, and teachers and parents should be aware of it so they can take early prevention (4).

It is believed that social, school, and family environments have an important influence on individuals’ behavior and psychological development (5). Middle school students can develop deviant behaviors in their daily academic life due to the influence of complex factors such as the environment, and many bullying behaviors are caused by such deviant behaviors (6). A bad environmental system can easily cause individuals to develop deviant behaviors, while a good environment can promote better development. It is important to pay attention to the influence of the external environment of society, school, and family on the growth process of adolescents, especially as school bullying is a group process and occurs more often among classmates (7). For middle school students, school is one of their main places of activity. According to a Unesco report (2018), environmental factors such as students’ low sense of belonging to campus, poor discipline on campus, and teachers’ differential treatment are all triggers of school bullying. Specifically, when teenagers’ sense of control is threatened, individuals who experience school chaos are more likely to prefer the theme of attack than those who experience good school order, which may trigger their attack behavior (8). Therefore, the school environment can affect the occurrence of bullying. School bullying is a type of bad interpersonal interaction in the peer interaction process, involving every member of the peer group (9); therefore, the study on a school bullying intervention can start with class interventions.

Research on school bullying should not only focus on the group level, but also on the individual level. Middle school students are in the adolescent period of individual growth and development. Although their cognition tends to be mature, the development of thinking quality is contradictory and unstable, which is undoubtedly a challenge for them. During this period, a major task of middle school students is self-identity (10), and their sense of confusion about the future may be one of the reasons for the high incidence of school bullying in middle school. Therefore, cultivating positive internal qualities (such as grit) and enhancing individual adaptability can help reduce the occurrence of school bullying.

As a positive psychological quality, grit refers to the perseverance and passion that an individual has to achieve their long-term goals and includes two dimensions: perseverance of efforts and consistency of interest (11). The quality of grit can not only effectively reduce the adverse effects of risk factors on individuals’ body and mind but also promote the healthy development of individuals (12–14); it can also prevent youth from engaging in criminal behavior in low-income areas (15). Grit not only plays a role in a good environment, but it also plays a positive regulatory role when an individual is hit or in a negative state. Individuals with high levels of grit tend to adapt to difficulties, such as persistent efforts and developing better problem-solving strategies (16). The study by Cui and Lan (17) also confirmed this point. For adolescent boys with higher levels of grit, harsh parenting did not affect aggressive behavior. In addition, grit also buffered the association between peer attachment and problem behavior among adolescent immigrants (18). It has been shown (19) that individuals with high levels of grit have a more active lifestyle that helps them to combat stress and have higher levels of psychological wellbeing during COVID-19. Grit is a protective personality trait that assists individuals in coping with risk and promotes the development of self-resilience (20). Individuals with high grit are more adept at self-emotional regulation, showing fewer negative emotions and more positive emotions and expectations, even in difficult situations such as failure, and try to beat their best (21). They also have stronger interpersonal and emotional resilience and are relatively less dependent on the Internet (22). Being in a stressful environment for an extended period can lead to psychological states, such as anxiety, depression, and tension, and individuals may seek inappropriate outlets in order to alleviate such psychological states (23). Ninth-graders with high grit are less likely to engage in problematic behaviors, such as drug use, running away from home, and fighting (24). Adolescents with higher levels of grit have higher self-control of emotions, behaviors, and thinking and higher self-efficacy, while those with lower levels of perseverance have lower self-control of their emotions, behaviors, and thinking and lower self-efficacy (25).Therefore, the cultivation of grit quality is beneficial to the individual’s physical and mental health.

Although grit is considered to be a relatively stable trait, it is also malleable (26). Mastery goals are related to the acquisition of knowledge and the development of skills (27), viewing learning as an end in itself, focusing on the development of one’s own abilities and on the understanding and mastery of tasks. Curriculum intervention can cultivate and enhance students’ grit, thus improving their academic performance (28). Therefore, based on previous studies, we designed and developed a series of grit training courses that adopted the whole-class intervention method and organically combined the group level with the individual level, so as to explore the internal relationship between school bullying and the grit of middle school students, and reveal the role of grit training in the prevention and intervention of school bullying.

## Materials and methods

### Participants

In this study, a total of 370 students from grade 1 and grade 2 in a central school in Changyang County, Hubei Province, were selected as participants by the cluster sampling method in 2020. The experimental group had two classes in grade 7 and two classes in grade 8. The control group also had two classes in grade 7 and two classes in grade 8, which were different classes from the experimental group. Taking the whole class as a unit to conduct a unified test, 364 valid questionnaires were obtained with an effective recovery rate of 98% after eliminating invalid questionnaires.

### Research method

#### Traditional bullying and traditional being bullied scales

This study adopts the “Middle School Student Bullying Scale,” which was used in the paper by Yang (2014) (29). The scale consists of 14 items, including seven questions on bullying and seven questions on being bullied. For example, “Others bully me in school” and “I join with my peers to exclude others.” The questionnaire was scored as 5 points, including 1 point for “none at all,” 2 points for “once or twice within half a year,” 3 points for “2–3 times a month,” 4 points for “once a week,” and 5 points for “several times a week.” The highest score is 35 points and the lowest score is 7 points. The higher the item score, the higher the degree of bullying or being bullied.

The Cronbach's α coefficient of the traditional bullying scale is 0.82, and the Cronbach's α coefficient of the traditional being bullied scale is 0.86, both of which have good reliability. At the same time, a confirmatory factor analysis found that the two scale models fit within acceptable range (χ^2^/*df *= 3.57, comparative fit index (CFI) = 0.99, Tucker-Lewis index (TLI) = 0.99, root-mean-square error of approximation (RMSEA) = 0.04; χ^2^/*df *= 20.11, CFI = 0.93, TLI = 0.90, RMSEA = 0.07).

#### Cyber bullying scale

The study used the “European Cyberbullying Intervention Scale (ECIPQ)” developed by Del Rey et al. (30). For example, “I threaten people through text or online messages.” There are 11 questions in the scale with a score of 5 points each. The highest score is 55 points and the lowest score is 11 points. The higher the score, the higher the degree of bullying. The Cronbach's α coefficient of the scale was 0.81 and a confirmatory factor analysis (χ^2^/*df *= 1.81, CFI = 0.99, TLI = 0.99, RMSEA = 0.03) was carried out, indicating that the scale had good reliability and validity.

#### Cyber being bullied scales

This study used the Youth Cyber bullying Scale (CSAC) developed by Veiga Simão (2017) (31). For example, “Someone is spreading rumors about me on the Internet.” The scale consists of six questions with a score of 1–5 points each. The highest score is 30 points and the lowest score is 6 points. The higher the score, the greater the degree of bullying. The Cronbach's α coefficient of this scale is 0.84. The model fitting results were acceptable by a confirmatory factor analysis (χ^2^/*df *= 2.54, CFI = 0.95, TLI = 0.91, RMSEA = 0.08).

#### Grit-S Scale

In this study, the Grit-S Scale, revised by Duckworth and Quinn (32), contains a total of eight questions, including four items from the two themes of “perseverance” and “consistency of interest,” and all items of consistency of interest were scored in reverse. Each question had a score of 1–5 points from “not like me at all” to “like me very much.” The higher the score, the higher the level of determination. For example, “I used to spend years accomplishing a goal” and “every few months I would get interested in something new and a goal.” The Cronbach's α coefficient of the scale was 0.79, and a confirmatory factor analysis (χ^2^/*df *= 4.81, CFI = 0.97, TLI = 0.95, RMSEA = 0.05) showed that the scale had good reliability and validity.

### The design of experiments

#### Intervention method

This study designed six intervention courses, one class of 45 min per week, as shown in Table 1.

**Table 1:** Intervention design of study 2.

Group	Pre-test (T1)	Intervention	Post-test (T2)
Experimental group	A1	X	A2
Control group	B1		B2

#### Curriculum intervention program

The theme of the first class was “I know what bullying is,” to introduce the concept of school bullying to students. Through the form of refuting text (33), four judgment questions related to school bullying were proposed, and the answers to the questions were in some contrast with daily cognition; for example, “fighting is a kind of school bullying” and the answer was “wrong.” This was in order to increase the students' interest in participating in class, and deepen the students' understanding of the concept of school bullying.

Lessons 2–6 introduced and developed grit. Grit mainly includes the two themes of interest consistency and unremitting efforts. Five courses were designed based on Duckworth's (2017) (34) four aspects of how to become a resolute person: “tracing inner passion,” “deliberately practicing to achieve joyful flow experience,” “life's call to duty,” and “learning to cope with failure.”

These six courses all include warm-up activities, teaching of theoretical knowledge, activity experience, and other characteristics of mental health education courses. Meanwhile, the content is presented in the form of an interactive teacher–student discussion, student discussion, group discussion, and so on. Approximately 10 min of audio mindfulness training is conducted before each class (35), so that students can transfer their attention to themselves, feel themselves, and enter the class in a relaxed state, which can be regarded as behavioral grit training. Approximately 5 min before the end of each class, the teacher will summarize and review the class to further deepen the students’ cognition.

### Executing processes

The test was carried out in a class, and the questionnaire was distributed and collected on the spot by the graduate students conducting the experiment. The principle of research confidentiality was explained. In addition to presenting the definition of school bullying in the questionnaire, the definition of school bullying was explained by the graduate students before the questionnaire was issued. One month later, the intervention program was carried out in the experimental class but not in the control class. Two months later, the post-test questionnaire was distributed and collected. The content of the post-test questionnaire was the same as the test questionnaire before the intervention, but the order of the scale was disrupted to avoid the practice effect. SPSS version 25.0 was used to input and analyze the data.

### Testing and data statistics

Taking the class as the unit, the level of bullying and grit of the participants in this study was investigated. The test was conducted by the trained graduate students under the uniform instruction text. The test lasted 40 min, and the questionnaire was collected on the spot after the test. SPSS version 25.0 was used for the data analysis.

## Results

### Correlation analysis between school bullying and persistence

The correlation analysis of participants’ grit and school bullying score showed a significantly negative correlation between grit and traditional bullying, traditional being bullied, cyber bullying, and cyber being bullied. There was a significant negative correlation between interest consistency, persistent efforts and traditional bullying, traditional being bullied, cyber bullying, and cyber being bullied. There was a significant positive correlation between traditional bullying, traditional being bullied, cyber bullying, and cyber being bullied (see Table 2).

**Table 2:** Correlation analysis between persistence and school bullying (pre-test data) (*N* = 364).

Item	1	2	3	4	5	6	7
Grit	1						
Consistency of interest	0.85**	1					
Unrelenting efforts	0.82**	0.40**	1				
Traditional Bullying	−0.15**	−0.08**	−0.09**	1			
Traditional being bullied	−0.13**	−0.12**	−0.09**	0.36**	1		
Cyber bullying	−0.13**	−0.15**	−0.07**	0.63**	0.28**	1	
Cyber being bullied	−0.11**	−0.16**	−0.02	0.32**	0.43**	0.47**	1

1 = Grit, 2 = Consistency of interest, 3 = Unrelenting efforts, 4 = Traditional Bullying, 5 = Traditional being bullied, 6 = Cyber bullying, 7 = Cyber being bullied.

* *p* < 0.05; **p < 0.01.

### Test of intervention effect

The grit course is proven to be effective as the grit scores changed before and after the test. The course improved the grit in the experimental group but the grit scores decreased in the control group. The results are shown in Table 3.

**Table 3:** Grit scores of pre-test (T1) and post-test (T2) for experimental group and control group.

Group	Pre-test (T1) (M ± SD)	Post-test (T2) (M ± SD)	*t*
Grit (control group)	3.44 ± 0.60	3.23 ± 0.82	3.67***
Grit (experimental group)	3.31 ± 0.94	3.40 ± 0.76	−1.17

***p < 0.001.

A paired sample t-test was conducted for the experimental group and the control group on the whole, traditional bullying, traditional being bullied, cyber bullying, and cyber being bullied. The results are shown in Table 4.

**Table 4:** Paired *t*-test of per-test (T1) and post-test (T2) for experimental group and control group.

	Group	*n*	Pre-test (T1)		Post-test (T2)		*t*
			M	SD	M	SD	
Traditional bullying	Experimental group	162	7.73	2.54	7.40	1.06	1.78
	Control group	200	7.54	1.78	7.74	1.93	−1.50
Traditional being bullied	Experimental group	159	10.46	5.10	9.65	3.55	2.49*
	Control group	199	9.63	3.87	10.58	5.21	−3.32**
Cyber bullying	Experimental group	160	12.27	3.33	11.94	2.03	1.71
	Control group	201	11.78	2.09	12.16	2.55	−2.77**
Cyber being bullied	Experimental group	162	7.52	3.44	6.96	1.82	2.78*
	Control group	201	7.01	2.19	7.40	2.46	−2.18*

* *p* < 0.05; **p < 0.01.

As can be seen from Table 4, the post-test data (T2) of traditional being bullied and cyber being bullied in the experimental group showed a significant decline compared with the pre-test data (T1). The post-test data (T2) of traditional bullying and cyber bullying also showed a downward trend. In the control group, the traditional bullying, traditional being bullied, cyber bullying, and cyber being bullied post-test data (T2) showed an upward trend, and the traditional being bullied, cyber bullying, and cyber being bullied post-test data (T2) significantly increased compared with the pre-test data (T1). The pre-test (T1) and post-test (T2) data of the experimental group and the control group showed significant changes in direction, which further verified the effectiveness of persistence theme curriculum intervention.

## Discussion

Although previous studies have shown that grit can promote the development of adaptive function (36, 37), there are few studies on the relationship between persistence and bullying, and even fewer empirical studies on the cultivation of persistence. Therefore, this study attempts to explore the relationship between grit and bullying under the framework of ecosystem theory and mastery goal theory, and further verify the effectiveness of mental health education courses with grit as the theme. The results show that a grit course intervention can effectively reduce the incidence of school bullying, which means cultivating and improving individual grit quality can effectively reduce incidents of school bullying.

The analysis discovered that grit was negatively correlated with traditional bullying, traditional being bullied, cyber bullying, and cyber being bullied, indicating that the higher the level of grit of middle school students, the lower the possibility of bullying and being bullied. Grit is negatively correlated with bullying and being bullied, which may be because individuals with high levels of grit are more likely to be attracted to activities they are interested in, and actively seek goals and meaning during this period (16), forming a better plan for their future. Moreover, individuals with high levels of grit have stronger self-control and higher self-discipline (11), which reduces the possibility of engaging in aggressive behaviors at school (38). Similarly, an experience of bullying will also have a negative impact on an individual’s mental health. Even the mild involvement of bullying victims will have a relatively serious negative impact (39), such as internalization problems like depression, anxiety (40), and psychological stress (41); the decline in grit will not be an exception. Individuals involved in high numbers of bullying incidents tend to spend most of their time and space occupied by the bully and the aftermath of the incident, lack or refuse to engage with other favorable external environments, and do not pay positive attention to their own development and growth, resulting in lower levels of persistence.

Both bullying and being bullied declined through the course intervention. The results show that the course intervention with the theme of “persistence” can improve the level of persistence of middle school students, and then reduce the possibility of individuals participating in bullying. As can be seen from the results, there was a significant decrease in the number of bullies in class 803. An effective intervention for bullies may be the focus of grit programs on cultivating one's inner passions and exploring one's own interests, allowing students to turn their attention to themselves and actively seek new goals or acquire new skills. When this goal is recognized, they show greater interest and grit in this goal, even when others give up. This is consistent with the theory of mastering goals. Goal-oriented students are more focused on actively learning new skills, striving to master skills or achieve goals, and improving skills (42). In addition, the cultivation of grit promoted the former bullies’ ability to control themselves (43) and inhibited their impulsiveness and aggression (38, 44); and the bullying behavior was correspondingly reduced (45, 46).

Although the curriculum intervention had some effect on both bullying and being bullied, it had a more significant effect on reducing bullying. The results showed that there were significantly fewer bullies in the experimental group after the intervention than before. This may be because in the course activities, students found classmates with similar interests and hobbies, formed good peer relationships, and helped individuals to obtain a higher sense of belonging (47). This helped them to feel more positive energy and support from others (48), increased their sense of hope (49), provided them with more psychological power to fight against bullying, and broke the imbalance of psychological power of bullying.

Later courses emphasize the positive response to a negative environment. Persistence, as a positive character, can reduce the negative impact of risk factors on individuals in an adverse environment, thus playing a positive protective role (50). To equip individuals with psychological resilience (51), when confronted with difficulties, they have the courage to seek support and help from people around them, so as to change the situation of being bullied or reduce the probability of being bullied (13, 52). Therefore, persistence can be considered to be a potential protective factor for individuals and a booster on the way to growth (53), which can prevent and protect middle school students from participating in bullying incidents.

This study demonstrated that the cultivation of grit can effectively reduce bullying at the whole and individual levels by using a whole-class intervention approach. This will help fill in the relevant research on positive traits of adolescents and provide a meaningful reference for campus safety education. Since the participants in this study only come from one school, considering the regional differences in China, representative samples can be recruited nationwide for the curriculum intervention in the future.

The present study has some limitations. First, our study relied on self-report measures and may have some biases. Second, we gathered the data from teachers and students regarding their perception of the program by interview and received good feedback; however, we did not quantify this part. Third, the use of simple comparative analyses limits the interpretation. Fourth, this paper used a simple methodology to explain our findings, which met our current need, but a more complex methodology is needed for the interpretation. Future studies are needed to clarify these limitations.

## Conclusion

The grit course effectively reduced the traditional bullying behavior and cyber bullying behavior of middle school students, and significantly reduced the traditional bullying behavior of bullies and traditional being bullied behavior of victims.

## Data Availability

The raw data supporting the conclusions of this article will be made available by the authors, without undue reservation.
